# Crystal structures of 1,1′-bis­(carb­oxy­meth­yl)-4,4′-bipyridinium derivatives

**DOI:** 10.1107/S2056989024005127

**Published:** 2024-06-04

**Authors:** Hitoshi Kumagai, Satoshi Kawata, Nobuhiro Ogihara

**Affiliations:** a 41-1 Yokomichi, Nagakute, Aichi 480-1192, Japan; b Nanakuma Jonan-ku, Fukuoka 814-0180, Japan; Tokyo University of Science, Japan

**Keywords:** crystal structure, hydrogen bonding, viologen

## Abstract

The crystal structures of 2-[1′-(carb­oxy­meth­yl)-4,4′-bi­pyridine-1,1′-diium-1-yl]acetate tetra­fluoro­borate, C_14_H_13_N_2_O_4_^+^·BF_4_^−^ or (Hbcbpy)(BF_4_), and neutral 1,1′-bis­(carboxyl­atometh­yl)-4,4′-bi­pyridine-1,1′-diium (bcbpy), C_14_H_20_N_2_O_8_, are reported. The asymmetric unit of the (Hbcbpy)(BF_4_) consists of a Hbcbpy^+^ monocation, a BF_4_^−^ anion, and one-half of a water mol­ecule. The BF_4_^−^ anion is disordered. Two pyridinium rings of the Hbcbpy^+^ monocation are twisted at a torsion angle of 30.3 (2)° with respect to each other. The Hbcbpy monocation contains a carb­oxy­lic acid group and a deprotonated carboxyl­ate group. Both groups exhibit both a long and a short C—O bond. The cations are linked by inter­molecular hydrogen-bonding inter­actions between the carb­oxy­lic acid and the deprotonated carboxyl­ate group to give one-dimensional zigzag chains. The asymmetric unit of the neutral bcbpy consists of one-half of the bcbpy and two water mol­ecules. In contrast to the Hbcbpy^+^ monocation, the neutral bcbpy mol­ecule contains two pyridinium rings that are coplanar with each other and a carboxyl­ate group with similar C—O bond lengths. The mol­ecules are connected by inter­molecular hydrogen-bonding inter­actions between water mol­ecules and carboxyl­ate groups, forming a three-dimensional hydrogen-bonding network.

## Abstract 2

The crystal structures of 2-[1′-(carb­oxy­meth­yl)-4,4′-bi­pyridine-1,1′-diium-1-yl]acetate tetra­fluoro­borate, C_14_H_13_N_2_O_4_^+^·BF_4_^−^ or (Hbcbpy)(BF_4_), and neutral 1,1′-bis­(carboxyl­atometh­yl)-4,4′-bi­pyridine-1,1′-diium (bcbpy), C_14_H_20_N_2_O_8_, are reported.

## 1. Chemical context

Viologen derivatives (*N*,*N*′-disubstituted bipyridinium salts) have been widely researched because of their reversible electroactivity, good photochromic properties, and high biochemical activity. Because of these inter­esting physical and chemical properties, the synthesis of organic polymers or metal–organic frameworks by assembling viologen derivatives using covalent bonds or coordination bonds is attracting attention (Jouhara *et al.*, 2019 ▸; Madasamy *et al.*, 2019 ▸; Sun & Zhang, 2015 ▸). Non-covalent inter­actions such as hydrogen-bonding and electrostatic inter­actions have been used to assemble functional mol­ecules in the field of crystal engin­eering (Desiraju, 2001 ▸; Horiuchi *et al.*, 2007 ▸; Lehn, 1995 ▸). We have been working on hydrogen-bonded assemblies to synthesize functional materials and have previously reported hydrogen-bonded assemblies in which functional mol­ecules such as redox-active ferrocene derivatives or tetra­thia­fulvalene are used (Kitagawa & Kawata, 2002 ▸; Nagayoshi *et al.*, 2003 ▸). In the present study, we focus on hydrogen-bonded assemblies of *N,N′*-bis­(2-carb­oxy­eth­yl)-4,4′-bipyridinium derivatives.
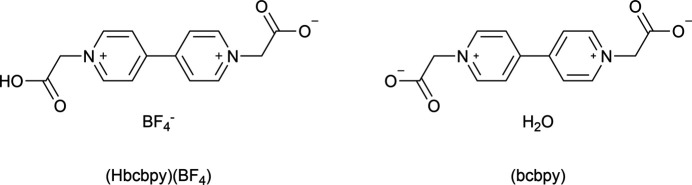


## 2. Structural commentary

**Figure 1:**
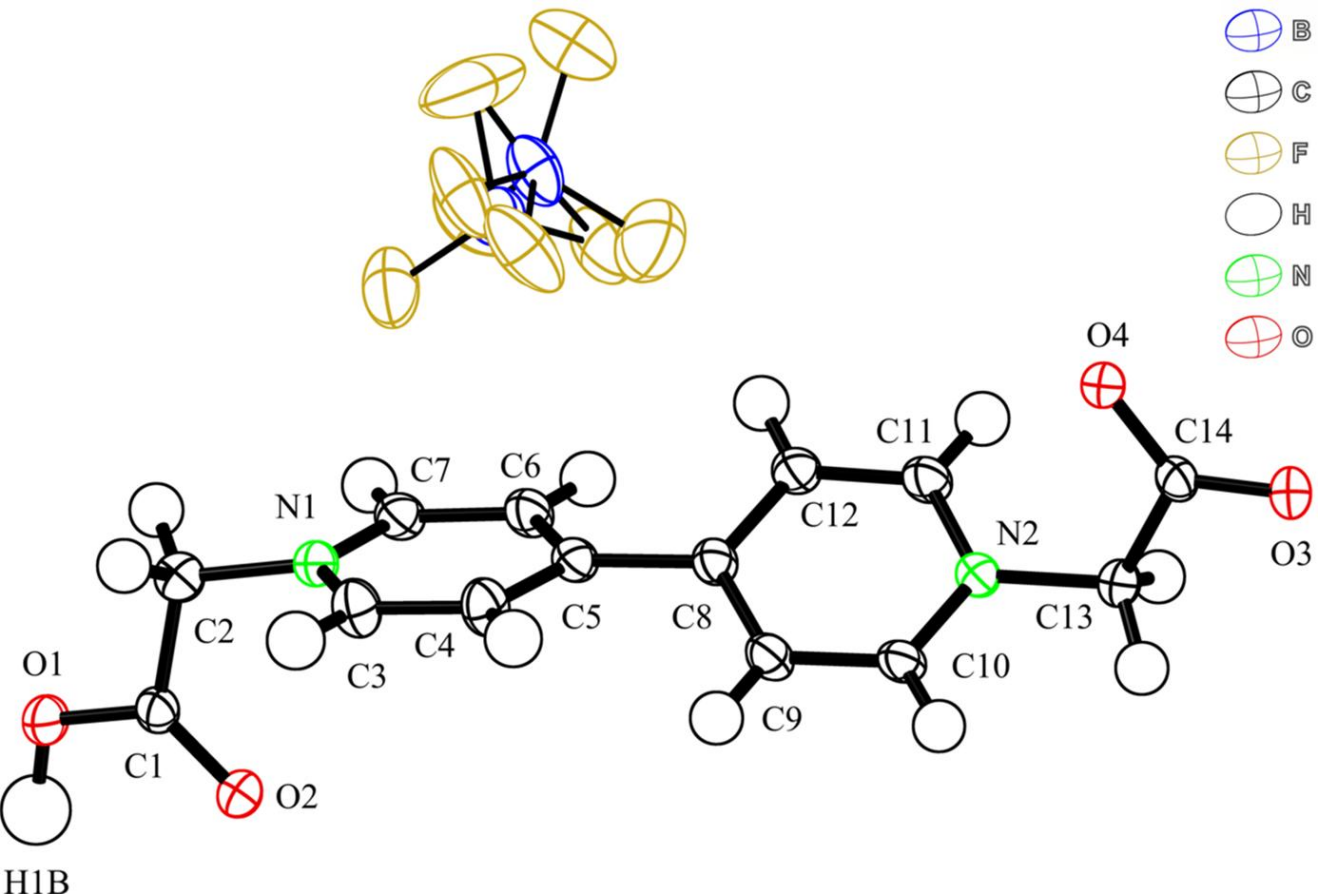
Structure of (Hbcbpy)(BF_4_) with labeling scheme and 50% probability displacement ellipsoids.

**Figure 2:**
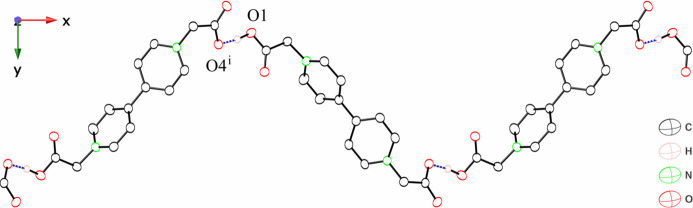
View of the hydrogen-bonded zigzag chain structure of Hbcbpy^+^ monocations. Dashed lines represent hydrogen bonds between oxygen atoms of Hbcbpy^+^ monocations. Only the carb­oxy­lic hydrogen atoms are shown for clarity. [Symmetry code: (i) *x* − 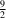, −*y* − 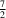, *z* − 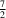.]

The asymmetric unit of (Hbcbpy)(BF_4_) consists of a Hbcbpy^+^ monocation, a BF_4_^−^ anion, and one-half of a water mol­ecule (Fig. 1 ▸). The BF_4_^−^ anion is disordered. The key feature of the structure is a hydrogen-bonded one-dimensional chain structure in which the chains are connected by inter­molecular –COO^−^⋯HOOC– hydrogen-bonding inter­actions (Table 1 ▸, Fig. 2 ▸). Because the water mol­ecules of (Hbcbpy)(BF_4_) are not involved in the hydrogen-bonding inter­actions, water mol­ecules are easily lost from the crystal to give partial occupancy. Two pyridinium groups of the Hbcbpy^+^ monocation are twisted at a C4—C5—C8—C9 torsion angle of 30.3 (2)° [dihedral angle between the rings = 30.18 (8)°] to each other. The carb­oxy­methyl groups bonded to the pyridinium groups exhibit a bent structure and are nearly perpendicular to the pyridinium groups. The Hbcbpy^+^ monocation contains a carb­oxy­lic acid group, –COOH, and a deprotonated negatively charged carboxyl­ate group, –COO^−^, at each end of the monocation. The charge is compensated by a BF_4_^−^ anion. The C—O and C=O bond lengths in the carb­oxy­lic acid group, C1—O1 and C1—O2, are 1.294 (2) and 1.223 (2) Å, respectively, with a difference of 0.071 (2) Å. Although the carboxyl­ate group is deprotonated, C14—O3 and C14—O4 also show two different bond lengths of 1.235 (2) and 1.287 (2) Å, respectively, where the difference is 0.052 (2) Å. The carb­oxy­lic acid group acts as a hydrogen-bond donor, and O4 of the deprotonated carboxyl­ate groups acts as a hydrogen-bond acceptor; the C14—O4 bond is longer than the C14—O3 bond. No hydrogen-bonding inter­actions are found for O3. The corresponding ClO_4_^−^ salt also exhibits two different C—O bond lengths in the deprotonated carboxyl­ate group and similar hydrogen-bonding inter­actions to give a zigzag chain structure (Gutov *et al.*, 2008 ▸). While the measurements of the (Hbcbpy)(BF_4_) were conducted at 100 K, the hydrogen-bonding distances of O(carb­oxy­lic acid)⋯O(carboxyl­ate) is very similar to that of the ClO_4_ salt measured at room temperature, indicative of a small influence of thermal libration (Gutov *et al.*, 2008 ▸). We assume that the different C—O bond lengths in the carboxyl­ate group arise from inter­molecular hydrogen-bonding inter­actions.

**Figure 3:**
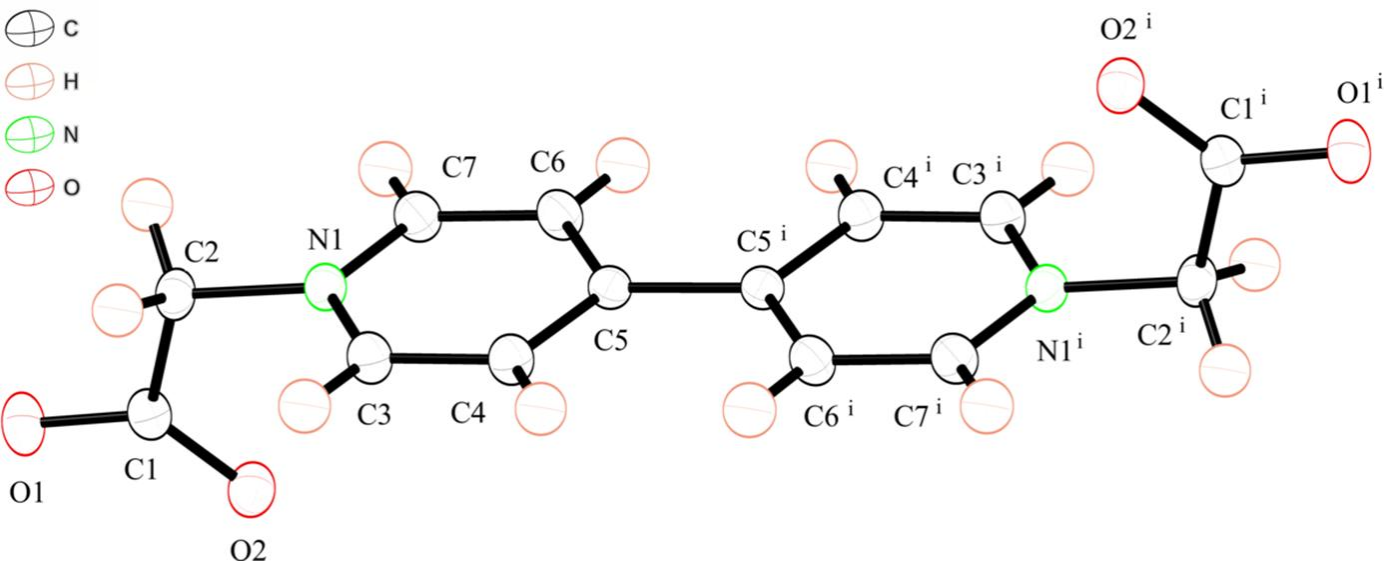
Structure of bcbpy with labeling scheme and 50% probability displacement ellipsoids. [Symmetry code: (i) *x* + 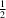, −*y* + 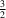, *z* + 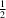]

**Figure 4:**
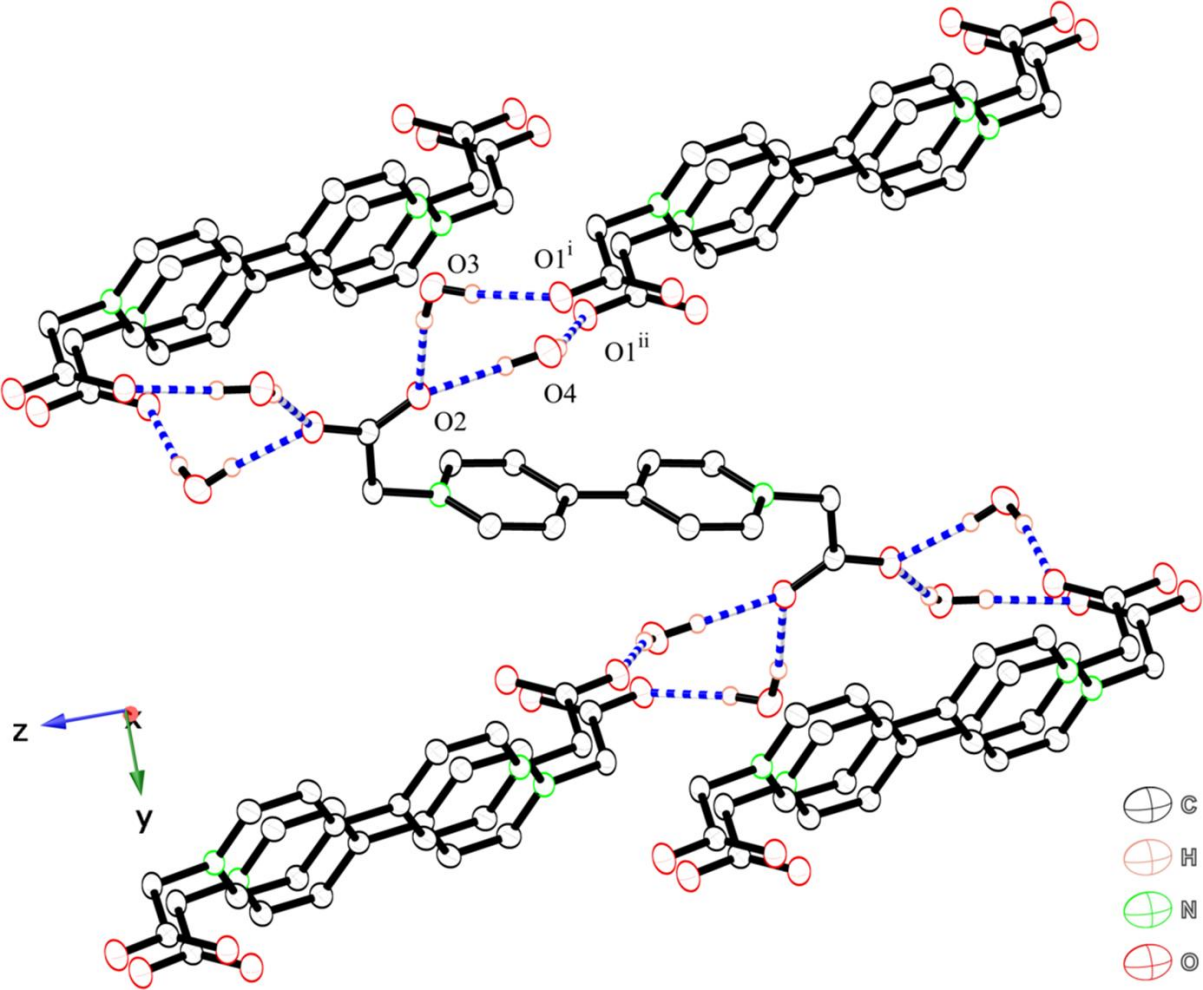
View of hydrogen-bonded network of bcbpy mol­ecules and water mol­ecules. Dashed lines represent hydrogen bonds between oxygen atoms. Hydrogen atoms of bcbpy are omitted for clarity. Only the hydrogen atoms of the water mol­ecules are shown for clarity. [Symmetry codes: (i) *x* + 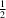, −*y* + 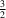, *z* + 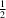; (ii) *x* − 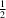, −*y* + 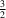, *z* + 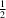.]

The asymmetric unit of bcbpy consists of one-half of the neutral bcbpy mol­ecule and two solvent water mol­ecules (Fig. 3 ▸). The key feature of the structure is a hydrogen-bonded three-dimensional network in which the mol­ecules are connected by inter­molecular hydrogen bonding inter­actions between bcbpy and water mol­ecules (Table 2 ▸, Fig. 4 ▸). In the bcbpy mol­ecule, two negatively charged deprotonated carboxyl­ate groups are attached to the pyridinium groups to give a neutral mol­ecule. The carb­oxy­methyl group bonded to the pyridinium group exhibits a structure similar to that of a Hbcbpy^+^ monocation. Two C—O bonds in the carboxyl­ate group form similar hydrogen-bonding inter­actions between water mol­ecules. Two differences are observed between the structure of the bcbpy mol­ecule and that of the Hbcbpy^+^ monocation. The first difference is the arrangement of the two pyridinium groups. Although the two pyridinium groups are twisted toward each other in the Hbcbpy^+^ monocation, they are coplanar with each other in the neutral bcbpy mol­ecule, with a C6—C5—C5^i^—C4^i^ torsion angle of 0.31 (14)°. The other difference is the C—O bond lengths in the deprotonated carboxyl­ate group. In the neutral bcbpy mol­ecule, the two C—O bond lengths are similar [1.248 (1) and 1.255 (1) Å, and the difference [0.007 (1) Å] is smaller than that between the C—O bond lengths in the deprotonated carboxyl­ate group in the Hbcbpy^+^ monocation. Both C—O bonds in the carboxyl­ate group undergo similar hydrogen-bonding inter­actions between water mol­ecules (Fig. 4 ▸). A neutral bcbpy mol­ecule without coordination bonds has been reported in [Zn(H_2_O)_6_]·(bcbpy)·(1,4-benzen di­carboxyl­ate)·3H_2_O (Zhao & Liu, 2021 ▸). The bcbpy mol­ecule in this Zn compound contains two types of carboxyl­ate groups. One carboxyl­ate group shows hydrogen-bonding inter­actions similar to those observed in our structure; the two C—O bond lengths in the carboxyl­ate group are 1.236 (3) and 1.249 (3) Å, differing by 0.013 (3) Å. The other carboxyl­ate group in the Zn compound exhibits two types of hydrogen-bonding inter­actions: inter­actions with three water mol­ecules, and an inter­action with one water mol­ecule. The two C—O bond lengths in the carboxyl­ate group are 1.235 (3) and 1.259 (3) Å; thus, the difference between the C—O bond lengths [0.024 (3) Å] is slightly larger than the corresponding difference for the first carboxyl­ate group. These results indicate that the difference between the two C—O bond lengths in the carboxyl­ate group is influenced by the type of hydrogen-bonding inter­actions in the bcbpy system.

## 3. Supra­molecular features

The Hbcbpy^+^ monocation contains a carb­oxy­lic acid and a deprotonated carboxyl­ate group at each end of the mono­cation. Inter­molecular hydrogen-bonding inter­actions occur between the carb­oxy­lic acid of one cation and the negatively charged carboxyl­ate groups of another monocation to give one-dimensional chains (Table 1 ▸, Fig. 2 ▸). The chains zigzag because of the bent structure of the carb­oxy­methyl groups attached to the pyridinium groups. Within the chains, the pyridinium rings are not coplanar, exhibiting no π–π stacking inter­actions.

The bcbpy mol­ecule contains two deprotonated carboxyl­ate groups at its ends. The negatively charged carboxyl­ate groups undergo inter­molecular hydrogen-bonding inter­actions between water mol­ecules (Table 2 ▸). The negatively charged carboxyl­ate groups act as hydrogen-bond acceptors, and water mol­ecules act as hydrogen-bond donors. The water mol­ecule bridges two bcbpy mol­ecules by hydrogen-bonding inter­actions, forming a three-dimensional hydrogen bonding network. Although the two pyridinium groups are coplanar, no π–π stacking inter­actions are observed.

Both compounds lack π–π stacking inter­actions. A possible explanation for this is that the carb­oxy­methyl groups bonded to the pyridinium groups, which are bent and nearly perpendicular to the pyridinium groups, prevent stacking inter­actions. Thus, the supra­molecular structures of the Hbcbpy^+^ monocation and bcbpy mol­ecule are primarily stabilized by the hydrogen-bonding inter­actions between negatively charged carboxyl­ate groups and carb­oxy­lic acids or water mol­ecules.

## 4. Database survey

A survey of the Cambridge Structural Database (CSD, v5.44, April 2023; Groom *et al.*, 2016 ▸) for structures with a bcbpy moiety resulted in nine matches. Of these, (Hbcbpy)(ClO_4_) (ODOQUV; Zhao & Liu, 2021 ▸) and (Zn(H_2_O)_6_)(bcbpy)(1,4-benzene di­carboxyl­ate) (AXEJEV; Gutov *et al.*, 2008 ▸) are related to the present work. Other compounds exhibit metal coordination and contain co-bridging ligands such as tri­carboxyl­ate or hexa­cyano metallate ions to compensate charges (Li *et al.*, 2020 ▸; Liu *et al.*, 2020 ▸; Ma *et al.*, 2009 ▸, 2011 ▸). The crystal structures of similar viologens in which carb­oxy­ethyl groups are attached to nitro­gen atoms have been reported, *viz*. 3-[1′-(2-carb­oxy­eth­yl)-4,4′-bipyridinium-1-yl]propano­ate perchlorate (ODOQOP; Gutov *et al.*, 2008 ▸) and *catena*-[tris­[1,1′-bis­(2-carb­oxy­eth­yl)-4,4′-bipyridinium]hexa­kis­(μ-bromo)­tri­bromo­trilead(II) dihydrate] (FEBLOQ; Sun *et al.*, 2017 ▸). ODOQOP is a monocation similar to ODOQUV. FEBLOQ is a dication in which [Pb_3_Br_9_]_*n*_^3*n*−^ chains act as counter-ions in the crystal. (4-{2-[1-(Carb­oxy­meth­yl)pyridin-1-ium-4-yl]ethen­yl}pyridin-1-ium-1-yl)acetate tetra­fluoro­bor­ate (MUPBUX), (4-{2-[1-(carb­oxy­meth­yl)pyridin-1-ium-4-yl]ethen­yl}pyridin-1-ium-1-yl)acetate perchlorate (MUPCAE), and tris­(aqua)-[1-(carb­oxy­meth­yl)-4-(2-{1-[(carb­oxy)meth­yl]pyridin-1-ium-4-yl}eth­en­yl)pyridin-1-iumato]lithium iodide dihydrate (MUPCEI) are ethyl­enic derivatives in which two pyridinium groups are linked by an ethyl­ene group (Jouhara *et al.*, 2019 ▸). MUPBUX and MUPCAE are monocations similar to the Hbcbpy^+^ monocation.

## 5. Synthesis and crystallization

The di­bromo salt H_2_bcbpy(Br)_2_ was synthesized using a modified version of a reported procedure (Fajardo & Lewis, 1997 ▸). The route is presented in the supporting information (scheme S1). (Hbcbpy)(BF_4_) and bcbpy were obtained as follows. An aqueous solution (50 mL) of Li(BF_4_) (38.6 g, 320 mmol) was added to an aqueous solution (50 mL) of H_2_bcbpy(Br)_2_ (20.0 g, 40 mmol). The mixture was stirred, the resultant white precipitate was collected by filtration, and the obtained solution was slowly evaporated to yield colorless crystals of (Hbcbpy)(BF_4_). The crystals (4.9 g, 11 mmol) were dissolved in 30 mL of distilled water, and an aqueous solution (20 mL) of LiOH (0.42 g, 18 mmol) was added. The resultant solution was evaporated, and the obtained white precipitate was filtered. Colorless crystals began to form from the obtained solution at ambient temperature. One of these crystals was used for X-ray crystallographic analysis.

## 6. Refinement

The crystal data, data collection, and structure refinement details are summarized in Table 3 ▸. The hydrogen atoms of the carb­oxy­lic acid and water mol­ecules, which are involved in hydrogen-bonding inter­actions were located in difference-Fourier maps and refined isotropically. Other hydrogen atoms were placed in idealized positions and refined using a riding model. The occupancy of the water mol­ecule in (Hbcbpy)(BF_4_) was refined.

## Supplementary Material

Crystal structure: contains datablock(s) global, HbcbpyBF4, bcbpy. DOI: 10.1107/S2056989024005127/jp2007sup1.cif

Structure factors: contains datablock(s) bcbpy. DOI: 10.1107/S2056989024005127/jp2007bcbpysup2.hkl

Supporting information file. DOI: 10.1107/S2056989024005127/jp2007bcbpysup4.cdx

Structure factors: contains datablock(s) HbcbpyBF4. DOI: 10.1107/S2056989024005127/jp2007HbcbpyBF4sup3.hkl

Supporting information file. DOI: 10.1107/S2056989024005127/jp2007HbcbpyBF4sup5.cdx

CCDC references: 2359279, 2359278

Additional supporting information:  crystallographic information; 3D view; checkCIF report

## Figures and Tables

**Table 1:** Hydrogen-bond geometry (Å, °) for (Hbcbpy)(BF_4_)

*D*—H⋯*A*	*D*—H	H⋯*A*	*D*⋯*A*	*D*—H⋯*A*
O1—H1*B*⋯O4^i^	1.05	1.42	2.4694 (18)	172.0

Symmetry code: (i) 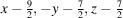.

**Table 2:** Hydrogen-bond geometry (Å, °) for bcbpy[Chem scheme1]

*D*—H⋯*A*	*D*—H	H⋯*A*	*D*⋯*A*	*D*—H⋯*A*
O3—H9⋯O2	0.859 (19)	1.96 (2)	2.8135 (12)	172.1 (19)
O3—H8⋯O1^i^	0.85 (2)	1.95 (2)	2.7888 (11)	171 (2)
O4—H10⋯O2	0.89 (2)	1.97 (2)	2.8233 (12)	158.9 (18)
O4—H11⋯O1^ii^	0.85 (2)	1.97 (2)	2.8007 (13)	164.1 (17)

Symmetry codes: (i) 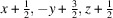; (ii) 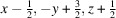.

**Table 3:** Experimental details

	(Hbcbpy)(BF_4_)	bcbpy
Crystal data		
Chemical formula	C_14_H_13_N_2_O_4.5_^+^·BF_4_^−^	C_14_H_20_N_2_O_8_
*M*_r_	368.08	344.32
Crystal system, space group	Monoclinic, *P*2_1_/*n*	Monoclinic, *P*2_1_/*n*
Temperature (K)	100	296
*a*, *b*, *c* (Å)	7.6794 (16), 20.987 (4), 10.0514 (19)	6.2521 (2), 11.4122 (4), 11.1413 (4)
β (°)	95.123 (3)	99.4832 (14)
*V* (Å^3^)	1613.5 (5)	784.07 (5)
*Z*	4	2
Radiation type	Mo *K*α	Mo *K*α
μ (mm^−1^)	0.14	0.12
Crystal size (mm)	0.60 × 0.40 × 0.40	0.48 × 0.46 × 0.42

Data collection		
Diffractometer	Rigaku R-AXIS RAPID	Rigaku R-AXIS RAPID
Absorption correction	Multi-scan (*ABSCOR*; Rigaku, 1995 ▸)	Multi-scan (*ABSCOR*; Rigaku, 1995 ▸)
*T*_min_, *T*_max_	0.656, 0.987	0.788, 0.951
No. of measured, independent and observed reflections	15631, 3652, 3119 [*I* > 2σ(*I*)]	7476, 1793, 1678 [*F*^2^ > 2.0σ(*F*^2^)]
*R*_int_	0.032	0.022
(sin θ/λ)_max_ (Å^−1^)	0.648	0.649

Refinement		
*R*[*F*^2^ > 2σ(*F*^2^)], *wR*(*F*^2^), *S*	0.051, 0.132, 1.05	0.048, 0.123, 1.11
No. of reflections	3652	1793
No. of parameters	292	125
No. of restraints	1	0
H-atom treatment	H atoms treated by a mixture of independent and constrained refinement	H atoms treated by a mixture of independent and constrained refinement
Δρ_max_, Δρ_min_ (e Å^−3^)	0.72, −0.56	0.40, −0.28

Computer programs: *RAPID-AUTO* (Rigaku, 1995 ▸), *Il Milione* (Burla *et al.*, 2007 ▸), *SUPERFLIP* (Palatinus & Chapuis, 2007 ▸), *SHELXL2018/3* (Sheldrick, 2015 ▸), *Yadokari-XG 2009* (Kabuto *et al.*, 2009 ▸) and *CrystalStructure* (Rigaku, 2019 ▸).

## supplementary crystallographic information

### 1,1'-Bis(carboxylatomethyl)-4,4'-bipyridine-1,1'-diium (bcbpy) Crystal data

**Table:**

C_14_H_20_N_2_O_8_	*F*(000) = 364.00
*M**_r_* = 344.32	*D*_x_ = 1.458 Mg m^−^^3^
Monoclinic, *P*2_1_/*n*	Mo *K*α radiation, λ = 0.71075 Å
*a* = 6.2521 (2) Å	Cell parameters from 7055 reflections
*b* = 11.4122 (4) Å	θ = 3.3–27.5°
*c* = 11.1413 (4) Å	µ = 0.12 mm^−^^1^
β = 99.4832 (14)°	*T* = 296 K
*V* = 784.07 (5) Å^3^	Block, yellow
*Z* = 2	0.48 × 0.46 × 0.42 mm

### 1,1'-Bis(carboxylatomethyl)-4,4'-bipyridine-1,1'-diium (bcbpy) Data collection

**Table:**

Rigaku R-AXIS RAPID diffractometer	1678 reflections with *F*^2^ > 2.0σ(*F*^2^)
Detector resolution: 10.000 pixels mm^-1^	*R*_int_ = 0.022
ω scans	θ_max_ = 27.5°, θ_min_ = 3.5°
Absorption correction: multi-scan (ABSCOR; Rigaku, 1995)	*h* = −8→7
*T*_min_ = 0.788, *T*_max_ = 0.951	*k* = −14→14
7476 measured reflections	*l* = −14→14
1793 independent reflections	

### 1,1'-Bis(carboxylatomethyl)-4,4'-bipyridine-1,1'-diium (bcbpy) Refinement

**Table:**

Refinement on *F*^2^	Secondary atom site location: difference Fourier map
*R*[*F*^2^ > 2σ(*F*^2^)] = 0.048	Hydrogen site location: inferred from neighbouring sites
*wR*(*F*^2^) = 0.123	H atoms treated by a mixture of independent and constrained refinement
*S* = 1.11	*w* = 1/[σ^2^(*F*_o_^2^) + (0.0813*P*)^2^ + 0.1761*P*] where *P* = (*F*_o_^2^ + 2*F*_c_^2^)/3
1793 reflections	(Δ/σ)_max_ < 0.001
125 parameters	Δρ_max_ = 0.40 e Å^−^^3^
0 restraints	Δρ_min_ = −0.28 e Å^−^^3^
Primary atom site location: structure-invariant direct methods	

### 1,1'-Bis(carboxylatomethyl)-4,4'-bipyridine-1,1'-diium (bcbpy) Special details

**Table:**

Geometry. All esds (except the esd in the dihedral angle between two l.s. planes) are estimated using the full covariance matrix. The cell esds are taken into account individually in the estimation of esds in distances, angles and torsion angles; correlations between esds in cell parameters are only used when they are defined by crystal symmetry. An approximate (isotropic) treatment of cell esds is used for estimating esds involving l.s. planes.
Refinement. Refinement was performed using all reflections. The weighted R-factor (wR) and goodness of fit (S) are based on F^2^. R-factor (gt) are based on F. The threshold expression of F^2^ > 2.0 sigma(F^2^) is used only for calculating R-factor (gt).

### 1,1'-Bis(carboxylatomethyl)-4,4'-bipyridine-1,1'-diium (bcbpy) Fractional atomic coordinates and isotropic or equivalent isotropic displacement parameters (Å^2^)

**Table:**

	*x*	*y*	*z*	*U*_iso_*/*U*_eq_	
O1	0.51613 (14)	0.68508 (8)	0.03041 (7)	0.0252 (2)	
O2	0.48570 (13)	0.70887 (7)	0.22699 (7)	0.0228 (2)	
O3	0.80684 (15)	0.87902 (8)	0.29910 (8)	0.0270 (2)	
O4	0.41417 (14)	0.74797 (8)	0.46745 (8)	0.0277 (2)	
N1	0.17838 (14)	0.54278 (8)	0.22070 (8)	0.0176 (2)	
C7	0.28870 (17)	0.46929 (10)	0.30354 (10)	0.0207 (3)	
H7	0.411238	0.430906	0.286205	0.025*	
C6	0.22108 (18)	0.45072 (9)	0.41363 (10)	0.0204 (3)	
H6	0.297168	0.399296	0.469856	0.025*	
C5	0.03839 (16)	0.50899 (9)	0.44103 (9)	0.0165 (3)	
C4	−0.07103 (17)	0.58473 (10)	0.35318 (9)	0.0194 (3)	
H4	−0.193261	0.624817	0.368421	0.023*	
C3	0.00173 (17)	0.60021 (10)	0.24390 (10)	0.0199 (3)	
H3	−0.071887	0.650736	0.185822	0.024*	
C2	0.26195 (17)	0.56648 (10)	0.10675 (9)	0.0195 (3)	
H2A	0.143316	0.591106	0.044479	0.023*	
H2B	0.322475	0.495057	0.079110	0.023*	
C1	0.43789 (17)	0.66260 (9)	0.12460 (9)	0.0183 (2)	
H9	0.709 (3)	0.8291 (18)	0.2700 (17)	0.041 (5)*	
H8	0.860 (3)	0.8538 (18)	0.369 (2)	0.049 (5)*	
H10	0.400 (3)	0.7365 (16)	0.387 (2)	0.041 (5)*	
H11	0.285 (3)	0.7551 (15)	0.4820 (18)	0.043 (5)*	

### 1,1'-Bis(carboxylatomethyl)-4,4'-bipyridine-1,1'-diium (bcbpy) Atomic displacement parameters (Å^2^)

**Table:**

	*U*^11^	*U*^22^	*U*^33^	*U*^12^	*U*^13^	*U*^23^
O1	0.0241 (4)	0.0337 (5)	0.0190 (4)	−0.0043 (3)	0.0071 (3)	0.0034 (3)
O2	0.0228 (4)	0.0264 (4)	0.0193 (4)	−0.0036 (3)	0.0037 (3)	−0.0010 (3)
O3	0.0265 (5)	0.0282 (5)	0.0259 (5)	−0.0060 (3)	0.0033 (3)	0.0055 (3)
O4	0.0210 (5)	0.0380 (5)	0.0241 (5)	0.0000 (3)	0.0041 (3)	−0.0065 (4)
N1	0.0185 (5)	0.0185 (4)	0.0162 (4)	−0.0025 (3)	0.0045 (3)	0.0000 (3)
C7	0.0206 (5)	0.0204 (5)	0.0221 (5)	0.0028 (4)	0.0067 (4)	0.0023 (4)
C6	0.0215 (5)	0.0198 (5)	0.0209 (5)	0.0032 (4)	0.0059 (4)	0.0039 (4)
C5	0.0173 (5)	0.0155 (5)	0.0171 (5)	−0.0023 (4)	0.0037 (4)	−0.0004 (4)
C4	0.0180 (5)	0.0212 (5)	0.0193 (5)	0.0016 (4)	0.0045 (4)	0.0013 (4)
C3	0.0191 (5)	0.0219 (5)	0.0186 (5)	0.0016 (4)	0.0029 (4)	0.0025 (4)
C2	0.0214 (5)	0.0230 (5)	0.0151 (5)	−0.0017 (4)	0.0058 (4)	−0.0003 (4)
C1	0.0162 (5)	0.0209 (5)	0.0182 (5)	0.0013 (4)	0.0035 (4)	0.0026 (4)

### 1,1'-Bis(carboxylatomethyl)-4,4'-bipyridine-1,1'-diium (bcbpy) Geometric parameters (Å, º)

**Table:**

O1—C1	1.2553 (13)	C6—C5	1.3982 (14)
O2—C1	1.2479 (14)	C6—H6	0.9300
O3—H9	0.86 (2)	C5—C4	1.3973 (15)
O3—H8	0.85 (2)	C5—C5^i^	1.4856 (19)
O4—H10	0.89 (2)	C4—C3	1.3794 (14)
O4—H11	0.85 (2)	C4—H4	0.9300
N1—C3	1.3454 (14)	C3—H3	0.9300
N1—C7	1.3495 (15)	C2—C1	1.5429 (15)
N1—C2	1.4753 (13)	C2—H2A	0.9700
C7—C6	1.3780 (14)	C2—H2B	0.9700
C7—H7	0.9300		
			
H9—O3—H8	105.7 (19)	C3—C4—H4	119.9
H10—O4—H11	105.3 (18)	C5—C4—H4	119.9
C3—N1—C7	120.97 (9)	N1—C3—C4	120.51 (10)
C3—N1—C2	119.62 (9)	N1—C3—H3	119.7
C7—N1—C2	119.27 (9)	C4—C3—H3	119.7
N1—C7—C6	120.47 (10)	N1—C2—C1	111.44 (8)
N1—C7—H7	119.8	N1—C2—H2A	109.3
C6—C7—H7	119.8	C1—C2—H2A	109.3
C7—C6—C5	120.17 (10)	N1—C2—H2B	109.3
C7—C6—H6	119.9	C1—C2—H2B	109.3
C5—C6—H6	119.9	H2A—C2—H2B	108.0
C4—C5—C6	117.68 (9)	O2—C1—O1	127.60 (10)
C4—C5—C5^i^	120.85 (11)	O2—C1—C2	118.49 (9)
C6—C5—C5^i^	121.47 (12)	O1—C1—C2	113.91 (9)
C3—C4—C5	120.19 (10)		
			
C3—N1—C7—C6	0.60 (17)	C7—N1—C3—C4	−0.25 (16)
C2—N1—C7—C6	176.33 (10)	C2—N1—C3—C4	−175.96 (10)
N1—C7—C6—C5	−0.68 (17)	C5—C4—C3—N1	−0.02 (17)
C7—C6—C5—C4	0.41 (16)	C3—N1—C2—C1	92.74 (11)
C7—C6—C5—C5^i^	−179.89 (11)	C7—N1—C2—C1	−83.05 (12)
C6—C5—C4—C3	−0.07 (16)	N1—C2—C1—O2	−1.12 (14)
C5^i^—C5—C4—C3	−179.76 (11)	N1—C2—C1—O1	179.23 (9)

Symmetry code: (i) −*x*, −*y*+1, −*z*+1.

### 1,1'-Bis(carboxylatomethyl)-4,4'-bipyridine-1,1'-diium (bcbpy) Hydrogen-bond geometry (Å, º)

**Table:**

*D*—H···*A*	*D*—H	H···*A*	*D*···*A*	*D*—H···*A*
O3—H9···O2	0.859 (19)	1.96 (2)	2.8135 (12)	172.1 (19)
O3—H8···O1^ii^	0.85 (2)	1.95 (2)	2.7888 (11)	171 (2)
O4—H10···O2	0.89 (2)	1.97 (2)	2.8233 (12)	158.9 (18)
O4—H11···O1^iii^	0.85 (2)	1.97 (2)	2.8007 (13)	164.1 (17)

Symmetry codes: (ii) *x*+1/2, −*y*+3/2, *z*+1/2; (iii) *x*−1/2, −*y*+3/2, *z*+1/2.

### 2-[1'-(Carboxymethyl)-4,4'-bipyridine-1,1'-diium-1-yl]acetate tetrafluoroborate (HbcbpyBF4) Crystal data

**Table:**

C_14_H_13_N_2_O_4.5_^+^·BF_4_^−^	*F*(000) = 756
*M**_r_* = 368.08	*D*_x_ = 1.519 Mg m^−^^3^
Monoclinic, *P*2_1_/*n*	Mo *K*α radiation, λ = 0.71075 Å
*a* = 7.6794 (16) Å	Cell parameters from 2543 reflections
*b* = 20.987 (4) Å	θ = 5.6–27.4°
*c* = 10.0514 (19) Å	µ = 0.14 mm^−^^1^
β = 95.123 (3)°	*T* = 100 K
*V* = 1613.5 (5) Å^3^	Platelet, colorless
*Z* = 4	0.60 × 0.40 × 0.40 mm

### 2-[1'-(Carboxymethyl)-4,4'-bipyridine-1,1'-diium-1-yl]acetate tetrafluoroborate (HbcbpyBF4) Data collection

**Table:**

Rigaku R-AXIS RAPID diffractometer	3119 reflections with *I* > 2σ(*I*)
Detector resolution: 10.000 pixels mm^-1^	*R*_int_ = 0.032
ω scans	θ_max_ = 27.4°, θ_min_ = 3.2°
Absorption correction: multi-scan (ABSCOR; Rigaku, 1995)	*h* = −9→9
*T*_min_ = 0.656, *T*_max_ = 0.987	*k* = −27→27
15631 measured reflections	*l* = −13→13
3652 independent reflections	

### 2-[1'-(Carboxymethyl)-4,4'-bipyridine-1,1'-diium-1-yl]acetate tetrafluoroborate (HbcbpyBF4) Refinement

**Table:**

Refinement on *F*^2^	Primary atom site location: structure-invariant direct methods
Least-squares matrix: full	Secondary atom site location: difference Fourier map
*R*[*F*^2^ > 2σ(*F*^2^)] = 0.051	Hydrogen site location: mixed
*wR*(*F*^2^) = 0.132	H atoms treated by a mixture of independent and constrained refinement
*S* = 1.05	*w* = 1/[σ^2^(*F*_o_^2^) + (0.0594*P*)^2^ + 1.2013*P*] where *P* = (*F*_o_^2^ + 2*F*_c_^2^)/3
3652 reflections	(Δ/σ)_max_ < 0.001
292 parameters	Δρ_max_ = 0.72 e Å^−^^3^
1 restraint	Δρ_min_ = −0.56 e Å^−^^3^

### 2-[1'-(Carboxymethyl)-4,4'-bipyridine-1,1'-diium-1-yl]acetate tetrafluoroborate (HbcbpyBF4) Special details

**Table:**

Geometry. All esds (except the esd in the dihedral angle between two l.s. planes) are estimated using the full covariance matrix. The cell esds are taken into account individually in the estimation of esds in distances, angles and torsion angles; correlations between esds in cell parameters are only used when they are defined by crystal symmetry. An approximate (isotropic) treatment of cell esds is used for estimating esds involving l.s. planes.

### 2-[1'-(Carboxymethyl)-4,4'-bipyridine-1,1'-diium-1-yl]acetate tetrafluoroborate (HbcbpyBF4) Fractional atomic coordinates and isotropic or equivalent isotropic displacement parameters (Å^2^)

**Table:**

	*x*	*y*	*z*	*U*_iso_*/*U*_eq_	Occ. (<1)
O4	−0.12434 (15)	0.59508 (5)	0.51560 (12)	0.0246 (3)	
O3	−0.18392 (15)	0.49606 (6)	0.58466 (13)	0.0278 (3)	
C14	−0.0977 (2)	0.54581 (7)	0.59083 (16)	0.0212 (3)	
C13	0.0546 (2)	0.54866 (8)	0.69960 (17)	0.0232 (3)	
H13	0.006964	0.550938	0.787824	0.028*	
H13A	0.123093	0.508798	0.697034	0.028*	
N2	0.17293 (17)	0.60349 (6)	0.68628 (13)	0.0206 (3)	
C10	0.3334 (2)	0.59435 (8)	0.64492 (17)	0.0224 (3)	
H10	0.368960	0.552814	0.621280	0.027*	
C9	0.4466 (2)	0.64527 (8)	0.63679 (17)	0.0233 (3)	
H9	0.559646	0.638678	0.607859	0.028*	
C8	0.3941 (2)	0.70639 (8)	0.67125 (16)	0.0211 (3)	
C5	0.5167 (2)	0.76126 (8)	0.67427 (16)	0.0221 (3)	
N1	0.75008 (18)	0.86160 (6)	0.68917 (14)	0.0227 (3)	
C12	0.2245 (2)	0.71446 (8)	0.70921 (17)	0.0249 (4)	
H12	0.183872	0.755753	0.729713	0.030*	
C7	0.6207 (2)	0.86054 (8)	0.77144 (18)	0.0252 (3)	
H7	0.611023	0.894042	0.833916	0.030*	
C1	1.0355 (2)	0.88901 (7)	0.80414 (16)	0.0208 (3)	
O1	1.14297 (16)	0.93451 (6)	0.84102 (13)	0.0305 (3)	
O2	1.04698 (15)	0.83348 (5)	0.84064 (12)	0.0240 (3)	
C11	0.1169 (2)	0.66241 (8)	0.71677 (17)	0.0246 (3)	
H11	0.002372	0.667885	0.743615	0.029*	
C6	0.5025 (2)	0.81110 (8)	0.76520 (17)	0.0250 (3)	
H6	0.410968	0.810802	0.822826	0.030*	
C2	0.8844 (2)	0.91214 (8)	0.70692 (18)	0.0246 (3)	
H2	0.832748	0.951166	0.742328	0.029*	
H2A	0.927993	0.922573	0.619744	0.029*	
F1A	0.2612 (3)	0.83677 (18)	0.3422 (3)	0.0623 (9)	0.662 (3)
C4	0.6512 (2)	0.76409 (8)	0.58975 (17)	0.0272 (4)	
H4	0.663465	0.731336	0.526009	0.033*	
F2A	0.1745 (3)	0.84329 (14)	0.5476 (2)	0.0524 (7)	0.662 (3)
C3	0.7663 (2)	0.81476 (9)	0.59933 (18)	0.0283 (4)	
H3	0.858056	0.816631	0.541966	0.034*	
B1A	0.2445 (7)	0.8769 (2)	0.4484 (4)	0.0336 (9)	0.662 (3)
F3A	0.4096 (3)	0.89999 (11)	0.4970 (2)	0.0540 (7)	0.662 (3)
F4A	0.1333 (5)	0.92664 (13)	0.4093 (3)	0.0938 (13)	0.662 (3)
B1B	0.1633 (13)	0.8772 (5)	0.4399 (9)	0.037 (2)	0.338 (3)
H1B	−0.259900	0.581900	0.413100	0.050*	
F1B	0.2530 (6)	0.9024 (3)	0.5528 (4)	0.0615 (15)	0.338 (3)
F2B	0.2596 (6)	0.8794 (4)	0.3323 (4)	0.0537 (15)	0.338 (3)
F3B	0.1320 (8)	0.8129 (3)	0.4697 (7)	0.0718 (17)	0.338 (3)
F4B	0.0007 (6)	0.9038 (2)	0.4137 (3)	0.0508 (13)	0.338 (3)
O5B	0.5838 (6)	0.9583 (2)	0.5161 (4)	0.0446 (11)	0.352 (2)
O5A	−0.1551 (12)	0.9609 (5)	0.4223 (9)	0.036 (2)	0.148 (2)

### 2-[1'-(Carboxymethyl)-4,4'-bipyridine-1,1'-diium-1-yl]acetate tetrafluoroborate (HbcbpyBF4) Atomic displacement parameters (Å^2^)

**Table:**

	*U*^11^	*U*^22^	*U*^33^	*U*^12^	*U*^13^	*U*^23^
O4	0.0198 (6)	0.0195 (6)	0.0340 (6)	−0.0016 (4)	−0.0005 (5)	0.0036 (5)
O3	0.0219 (6)	0.0218 (6)	0.0396 (7)	−0.0056 (5)	0.0020 (5)	0.0037 (5)
C14	0.0158 (7)	0.0200 (7)	0.0285 (8)	−0.0005 (6)	0.0057 (6)	−0.0001 (6)
C13	0.0203 (8)	0.0192 (7)	0.0303 (8)	−0.0010 (6)	0.0034 (6)	0.0037 (6)
N2	0.0172 (6)	0.0193 (6)	0.0251 (7)	−0.0003 (5)	0.0008 (5)	−0.0002 (5)
C10	0.0166 (7)	0.0206 (7)	0.0297 (8)	0.0028 (6)	0.0005 (6)	−0.0042 (6)
C9	0.0168 (7)	0.0238 (8)	0.0294 (8)	0.0001 (6)	0.0024 (6)	−0.0057 (6)
C8	0.0192 (7)	0.0204 (8)	0.0236 (7)	−0.0014 (6)	0.0002 (6)	−0.0026 (6)
C5	0.0183 (7)	0.0201 (7)	0.0273 (8)	−0.0001 (6)	−0.0016 (6)	−0.0016 (6)
N1	0.0200 (7)	0.0181 (6)	0.0294 (7)	−0.0015 (5)	−0.0019 (5)	−0.0004 (5)
C12	0.0213 (8)	0.0194 (8)	0.0339 (9)	0.0018 (6)	0.0022 (6)	−0.0047 (7)
C7	0.0216 (8)	0.0204 (8)	0.0330 (9)	0.0016 (6)	0.0005 (6)	−0.0059 (7)
C1	0.0185 (7)	0.0186 (7)	0.0260 (8)	−0.0003 (6)	0.0053 (6)	−0.0016 (6)
O1	0.0262 (6)	0.0195 (6)	0.0438 (7)	−0.0050 (5)	−0.0087 (5)	0.0016 (5)
O2	0.0231 (6)	0.0179 (6)	0.0311 (6)	−0.0008 (4)	0.0021 (5)	0.0012 (5)
C11	0.0190 (8)	0.0229 (8)	0.0322 (8)	0.0025 (6)	0.0046 (6)	−0.0045 (7)
C6	0.0198 (8)	0.0229 (8)	0.0325 (9)	0.0007 (6)	0.0041 (6)	−0.0048 (7)
C2	0.0216 (8)	0.0176 (7)	0.0338 (9)	−0.0031 (6)	−0.0011 (6)	0.0010 (6)
F1A	0.0304 (11)	0.099 (2)	0.0589 (15)	−0.0085 (14)	0.0125 (10)	−0.0396 (17)
C4	0.0267 (9)	0.0260 (8)	0.0291 (8)	−0.0052 (7)	0.0044 (7)	−0.0081 (7)
F2A	0.0353 (11)	0.0829 (18)	0.0405 (11)	−0.0104 (11)	0.0127 (9)	0.0096 (12)
C3	0.0265 (9)	0.0294 (9)	0.0294 (9)	−0.0051 (7)	0.0054 (7)	−0.0055 (7)
B1A	0.034 (2)	0.0352 (19)	0.032 (2)	0.000 (2)	0.007 (2)	0.0040 (14)
F3A	0.0532 (13)	0.0619 (14)	0.0449 (11)	−0.0261 (10)	−0.0073 (9)	0.0120 (10)
F4A	0.122 (3)	0.0458 (15)	0.103 (2)	0.0258 (17)	−0.054 (2)	0.0037 (14)
B1B	0.028 (4)	0.058 (5)	0.026 (4)	−0.009 (5)	0.011 (4)	−0.004 (3)
F1B	0.049 (3)	0.100 (4)	0.034 (2)	0.000 (2)	−0.0034 (17)	−0.020 (2)
F2B	0.033 (2)	0.103 (4)	0.0269 (19)	−0.027 (3)	0.0100 (15)	−0.006 (3)
F3B	0.075 (4)	0.059 (3)	0.085 (4)	−0.002 (3)	0.026 (3)	0.021 (3)
F4B	0.038 (2)	0.091 (3)	0.0237 (17)	0.009 (2)	0.0002 (14)	−0.0136 (18)
O5B	0.050 (3)	0.041 (2)	0.041 (2)	−0.004 (2)	−0.0052 (19)	0.0051 (19)
O5A	0.040 (5)	0.037 (5)	0.030 (4)	−0.001 (4)	0.000 (4)	0.002 (4)

### 2-[1'-(Carboxymethyl)-4,4'-bipyridine-1,1'-diium-1-yl]acetate tetrafluoroborate (HbcbpyBF4) Geometric parameters (Å, º)

**Table:**

O4—C14	1.287 (2)	C12—H12	0.9500
O4—H1B	1.4248	C7—C6	1.376 (2)
O3—C14	1.2350 (19)	C7—H7	0.9500
C14—C13	1.529 (2)	C1—O2	1.2226 (19)
C13—N2	1.480 (2)	C1—O1	1.2944 (19)
C13—H13	0.9900	C1—C2	1.528 (2)
C13—H13A	0.9900	C11—H11	0.9500
N2—C10	1.349 (2)	C6—H6	0.9500
N2—C11	1.353 (2)	C2—H2	0.9900
C10—C9	1.384 (2)	C2—H2A	0.9900
C10—H10	0.9500	F1A—B1A	1.375 (5)
C9—C8	1.398 (2)	C4—C3	1.381 (2)
C9—H9	0.9500	C4—H4	0.9500
C8—C12	1.399 (2)	F2A—B1A	1.370 (5)
C8—C5	1.486 (2)	C3—H3	0.9500
C5—C4	1.396 (2)	B1A—F4A	1.384 (5)
C5—C6	1.400 (2)	B1A—F3A	1.404 (6)
N1—C3	1.348 (2)	B1B—F2B	1.364 (9)
N1—C7	1.349 (2)	B1B—F4B	1.372 (12)
N1—C2	1.479 (2)	B1B—F1B	1.380 (10)
C12—C11	1.376 (2)	B1B—F3B	1.406 (11)
			
C14—O4—H1B	109.7	O2—C1—O1	126.19 (15)
O3—C14—O4	126.40 (15)	O2—C1—C2	121.66 (14)
O3—C14—C13	116.20 (14)	O1—C1—C2	112.15 (13)
O4—C14—C13	117.40 (14)	N2—C11—C12	120.56 (15)
N2—C13—C14	113.73 (13)	N2—C11—H11	119.7
N2—C13—H13	108.8	C12—C11—H11	119.7
C14—C13—H13	108.8	C7—C6—C5	120.26 (15)
N2—C13—H13A	108.8	C7—C6—H6	119.9
C14—C13—H13A	108.8	C5—C6—H6	119.9
H13—C13—H13A	107.7	N1—C2—C1	109.32 (13)
C10—N2—C11	121.13 (14)	N1—C2—H2	109.8
C10—N2—C13	120.19 (13)	C1—C2—H2	109.8
C11—N2—C13	118.68 (13)	N1—C2—H2A	109.8
N2—C10—C9	120.27 (15)	C1—C2—H2A	109.8
N2—C10—H10	119.9	H2—C2—H2A	108.3
C9—C10—H10	119.9	C3—C4—C5	119.62 (16)
C10—C9—C8	119.83 (14)	C3—C4—H4	120.2
C10—C9—H9	120.1	C5—C4—H4	120.2
C8—C9—H9	120.1	N1—C3—C4	120.75 (16)
C9—C8—C12	118.34 (15)	N1—C3—H3	119.6
C9—C8—C5	121.34 (14)	C4—C3—H3	119.6
C12—C8—C5	120.25 (14)	F2A—B1A—F1A	108.7 (4)
C4—C5—C6	118.07 (15)	F2A—B1A—F4A	108.8 (4)
C4—C5—C8	121.48 (15)	F1A—B1A—F4A	109.9 (4)
C6—C5—C8	120.43 (15)	F2A—B1A—F3A	109.0 (3)
C3—N1—C7	121.07 (14)	F1A—B1A—F3A	109.7 (4)
C3—N1—C2	119.89 (14)	F4A—B1A—F3A	110.7 (4)
C7—N1—C2	118.82 (14)	F2B—B1B—F4B	112.4 (7)
C11—C12—C8	119.80 (15)	F2B—B1B—F1B	111.9 (7)
C11—C12—H12	120.1	F4B—B1B—F1B	112.4 (7)
C8—C12—H12	120.1	F2B—B1B—F3B	108.5 (8)
N1—C7—C6	120.23 (15)	F4B—B1B—F3B	105.1 (7)
N1—C7—H7	119.9	F1B—B1B—F3B	106.0 (8)
C6—C7—H7	119.9		
			
O3—C14—C13—N2	168.93 (14)	C2—N1—C7—C6	−174.36 (15)
O4—C14—C13—N2	−11.5 (2)	C10—N2—C11—C12	−1.5 (2)
C14—C13—N2—C10	−107.12 (17)	C13—N2—C11—C12	178.25 (15)
C14—C13—N2—C11	73.10 (19)	C8—C12—C11—N2	−0.8 (3)
C11—N2—C10—C9	2.0 (2)	N1—C7—C6—C5	0.6 (3)
C13—N2—C10—C9	−177.78 (15)	C4—C5—C6—C7	−1.1 (2)
N2—C10—C9—C8	−0.2 (2)	C8—C5—C6—C7	177.33 (15)
C10—C9—C8—C12	−2.0 (2)	C3—N1—C2—C1	−84.48 (19)
C10—C9—C8—C5	175.02 (15)	C7—N1—C2—C1	90.16 (17)
C9—C8—C5—C4	30.3 (2)	O2—C1—C2—N1	10.3 (2)
C12—C8—C5—C4	−152.72 (17)	O1—C1—C2—N1	−169.22 (14)
C9—C8—C5—C6	−148.12 (17)	C6—C5—C4—C3	0.9 (3)
C12—C8—C5—C6	28.9 (2)	C8—C5—C4—C3	−177.53 (16)
C9—C8—C12—C11	2.5 (2)	C7—N1—C3—C4	−0.4 (3)
C5—C8—C12—C11	−174.60 (15)	C2—N1—C3—C4	174.09 (16)
C3—N1—C7—C6	0.2 (3)	C5—C4—C3—N1	−0.1 (3)

### 2-[1'-(Carboxymethyl)-4,4'-bipyridine-1,1'-diium-1-yl]acetate tetrafluoroborate (HbcbpyBF4) Hydrogen-bond geometry (Å, º)

**Table:**

*D*—H···*A*	*D*—H	H···*A*	*D*···*A*	*D*—H···*A*
C13—H13*A*···O1^i^	0.99	2.43	3.387 (2)	161
C10—H10···O3^ii^	0.95	2.61	3.126 (2)	114
C10—H10···O1^i^	0.95	2.51	3.362 (2)	149
C12—H12···O2^iii^	0.95	2.28	3.188 (2)	159
C7—H7···O3^iv^	0.95	2.34	3.208 (2)	151
C2—H2···O3^iv^	0.99	2.36	3.231 (2)	146
C4—H4···O2^v^	0.95	2.41	3.278 (2)	151
O4—H1*B*···O2^vi^	1.42	2.39	3.3044 (17)	118.1
O1—H1*B*···O4^vii^	1.05	1.42	2.4694 (18)	172.0

Symmetry codes: (i) −*x*+3/2, *y*−1/2, −*z*+3/2; (ii) −*x*, −*y*+1, −*z*+1; (iii) *x*−1, *y*, *z*; (iv) −*x*+1/2, *y*+1/2, −*z*+3/2; (v) *x*−1/2, −*y*+3/2, *z*−1/2; (vi) *x*−3/2, −*y*+3/2, *z*−1/2; (vii) *x*−9/2, −*y*−7/2, *z*−7/2.
